# Prioritization of Users in Ecosystem Service Valuation: Implications for the Design of Differentiated Conservation Policies

**DOI:** 10.1002/ece3.72344

**Published:** 2025-10-17

**Authors:** Blanca Isabel Sánchez Toledano, Mercedes Borja Bravo, Rafael García Vázquez, Marco Andrés López Santiago

**Affiliations:** ^1^ Campo Experimental Zacatecas Instituto Nacional de Investigaciones Forestales, Agrícolas y Pecuarias Zacatecas México; ^2^ Campo Experimental Pabellón Instituto Nacional de Investigaciones Forestales, Agrícolas y Pecuarias Aguascalientes México; ^3^ Universidad Autónoma Chapingo División de Ciencias Económico Administrativas Texcoco México; ^4^ Universidad Autónoma Chapingo Unidad Regional Universitaria de Zonas Áridas Bermejillo, Dgo México

**Keywords:** educational level, multivariate tools, public policies, strategies, valuation, variables

## Abstract

Ecosystem Services (ESs) valuation is a fundamental tool for understanding the interrelationships between society and the benefits provided by ecosystems, especially in contexts with specific territorial characteristics. This study aimed to analyze the relationship between the educational level and the prioritization of ESs in the Laguna de Bustillos basin, located in Cuauhtémoc, Chihuahua, Mexico, to provide elements for the design of differentiated and adaptive public policies aimed at ecosystem conservation. The methodology used consisted of the application of structured surveys to collect socioeconomic information and identify the presence and perceived value of provisioning, regulating, cultural, and supporting ESs. Data analysis was conducted using the nonparametric Kruskal–Wallis test and Principal Component Analysis (PCA). The results showed a higher prevalence of provisioning services (64%), followed by supporting (60%) and cultural services (59%), whereas regulating services were less prevalent (54%). Statistically significant differences were found in ES valuation according to users' educational level. The PCA revealed two structural components: one associated with provisioning and regulating services, and another related to cultural and supporting services. These findings support the proposal of differentiated conservation strategies aligned with the social priorities of various educational groups. It is concluded that public conservation policies must incorporate the diversity of social perceptions as an essential element for achieving sustainable and participatory environmental management.

## Graphical abstract

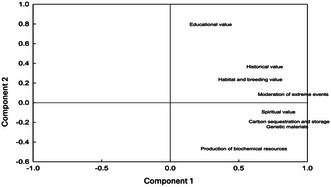

## 1 Introduction

Ecosystems are becoming increasingly vulnerable due to climate change and human activities (Li et al. 2021; Chen et al. 2023). In this context, environmental sustainability has become a global concern, leading to a growing recognition of the importance of conserving biodiversity and properly valuing Ecosystem Services (ESs) (Beillouin et al. 2021).

ESs have been defined by authors such as Constanza et al. (2014), Fang et al. (2021), Li et al. (2021), and Wang et al. (2022) as the set of benefits (both goods and services) that ecosystems provide to society, establishing close links between natural capital and social well‐being.

The valuation of ESs has been addressed from three main approaches: biophysical, which describes the ecological components and processes that generate them; monetary, which estimates their economic value and supports conservation investments; and sociocultural, which identifies values, attitudes, and meanings associated with the social demand for ESs (Zambrano et al. 2021).

Although an integrated approach is essential to promote resilient ecosystems, in practice biophysical and monetary approaches often prevail over sociocultural ones, thereby limiting the understanding of interactions between people and ecosystems (Zambrano et al. 2021). In this regard, the Millennium Ecosystem Assessment (MEA) (2005) proposed a widely accepted typology that classifies ESs into four categories: provisioning, regulating, cultural, and supporting services.

At the international level, the MEA marked a significant milestone by placing issues such as sustainability, biodiversity, conservation, and resource management on the global agenda (Wang et al. 2021). Subsequently, new approaches have expanded the analysis to include aspects such as cultural services, social perception, green infrastructure, and poverty alleviation (Wang et al. 2021).

However, the limited integration of environmental, economic, and social dimensions restricts the practical application of this knowledge (Mikusiński and Niedziałkowski 2020). Likewise, little attention has been paid to the different sociocultural factors that characterize territories (Sánchez et al. 2023).

In recent years, research on ESs has expanded rapidly, addressing the dynamic effects of supply and demand (Cao et al. 2025), the simulation of land‐use changes and their implications for ESs' value (Zhang et al. 2024), and the quantification of risks associated with food, drinking water, biodiversity, and recreation (Meraj et al. 2022).

Despite these advancements, the literature underscores the significant gaps between the potential of such assessments and their effective implementation in policy and management (Mikusiński and Niedziałkowski 2020). This highlights the urgent need to incorporate sociocultural dimensions into the ESs valuation (Mandle et al. 2021).

This need is supported by the recognition that sociodemographic factors such as educational level, gender, occupation, and cultural context influence the way people prioritize certain types of ESs (Janeczko et al. 2023; Pinto et al. 2023; Trujillo et al. 2023; Bhatt et al. 2024; Li et al. 2025). Moreover, the spatiotemporal variation in the perception of ESs constitutes a key factor for guiding sustainable conservation strategies (Pan et al. 2021).

Understanding these factors is crucial for designing legitimate and effective environmental policies (Zhang, Lu, et al. 2021; Li et al. 2025). By integrating socioeconomic variables, territorial policies can be strengthened and prioritized to align with the environmental values of different social groups (Janeczko et al. 2023).

For all these reasons, this study aimed to analyze the relationship between the educational level and the prioritization of ESs in the Laguna de Bustillos basin, Cuauhtémoc, Chihuahua, Mexico, to provide elements for the design of differentiated and adaptive public policies aimed at ecosystem conservation.

This research makes a novel contribution by demonstrating how educational level influences the valuation of MEA‐classified services (2005) in a semi‐arid region of northern Mexico, addressing the need for territorial studies that integrate social and cultural variables into ESs assessment.

## 2 Methodology

### 2.1 Study Area

The research was conducted in the municipality of Cuauhtémoc, Chihuahua, Mexico, which encompasses the Laguna Bustillos basin. The region is located within the geographical coordinates 28°38′51″ N—28°28′27″ N and 106°57′3″ W—106°38′50″ W (Rojas‐Villalobos et al. 2018).

The basin constitutes an endorheic system whose only water input comes from annual rainfall; it has an approximate area of 3298 km^2^, delimited by mountain formations, such as Pedernales, San Juan, Salitrera, Chuchupate, Sierra Azul, and Rebote (Leal et al. 2024), which shape the physical framework of this hydroecological region. The area climate is classified as warm and semi‐arid, typical of a region of ecological transition between the semi‐humid climate of the Sierra Madre Occidental and the arid conditions of the Chihuahua Desert (Rojas‐Villalobos et al. 2018).

In terms of land use, the area has a combination of pine‐oak forests, grasslands (natural and induced), halophytic vegetation, and agricultural land, both irrigated and rainfed. Pine‐oak forests predominate in mountainous areas, whereas grasslands extend over hills and foothill areas (Reyes et al. 2024).

From a biological perspective, the Laguna Bustillos is an area of ecological value. One hundred eighty‐seven species of fauna have been recorded there, highlighting its relevance as a key site for bird conservation in Mexico. In total, 25 species of birds have been identified, of which 16% are permanent residents, whereas 84% correspond to winter migratory birds (Reyes et al. 2024).

### 2.2 Sample Size Estimation

The information was collected through structured surveys applied in 2024. A representative sample was determined using the finite populations method, based on the population of the municipality of Cuauhtémoc, Chihuahua for the year 2020, according to the following formula (Borja et al. 2018; Téllez‐Delgado et al. 2012):

$$n=\frac{NZ^{2}\mathrm{pq}}{e^{2}\left(N-1\right)+Z^{2}\mathrm{pq}}$$

where *n* is the sample size; *N* is the total population of the municipality of Cuauhtémoc, Chihuahua (180,638 inhabitants) (National Institute of Statistics and Geography [INEGI] 2020); Z is the value of the standard normal distribution for a 90% confidence level (1.64); *p* is the probability of a representative sample (0.5); q is the probability of a not representative sample (1 − *p*); and e is the estimate's error, in this case 0.10 (10%). The estimated sample size was 67 people. To guarantee the representativeness of the different social groups present in the study area, the participants were selected through simple random sampling. In‐person surveys were conducted in homes surrounding the basin and in public spaces frequented by residents.

Although the institution lacks an institutional ethics committee, the ethical procedure was guaranteed by the informed consent of the respondents. Participation was entirely voluntary and anonymous, and all personal data were handled in accordance with the Ley Federal de Protección de Datos Personales en Posesión de los Particulares of Mexico (Federal Law on the Protection of Personal Data Held by Private Parties 2025). The law states that, in the case of sensitive personal data, the data controller must obtain the express written consent of the owner for its processing, through his autograph signature, electronic signature, or any authentication mechanism established for such purpose.

### 2.3 Instrument and Socioeconomic Variables

The survey applied in this study had two main purposes. The first was to collect socioeconomic information from the participants, including age, gender, education level, locality, and employment. The categories used were as follows:

- Age: (1) 20–30 years, (2) 31–40 years, (3) 41–50 years, (4) 51–60 years, (5) 61 years or older.
- Gender: (1) Male, (2) Female.
- Education level: (1) Primary education, (2) Secondary education, (3) High school, (4) University, (5) Postgraduate.
- Locality: (1) Rural communities surrounding the Laguna, (2) City of Cuauhtémoc, (3) Other localities.
- Employment: (1) Farmer or rancher, (2) Entrepreneur, (3) Employee, (4) Public servant, (5) Self‐employed, (6) Other activities.

### 2.4 ESs Valuation

The second purpose of the questionnaire was to identify respondents' perceptions of the ESs provided by the Laguna Bustillos basin. To this purpose, a list of ESs classified according to the typology proposed by MEA (2005) was included: provisioning, regulating, cultural, and supporting services (Table 1).

**TABLE 1:** Classification of ESs.

Provisioning services	Regulating services	Cultural services	Supporting services
Food production	Climate regulation	Recreational activities	Habitat and breeding place
Ornamental species production	Carbon sequestration and storage	Spiritual value	Nutrient cycling
Water storage and retention	Water flow regulation	Educational value	Primary productivity
Production of raw materials and fuels	Extreme event moderation	Historical value	
Biochemical resource production	Erosion control	Esthetic value	
Genetic materials	Biological regulation	Cultural heritage and identity	

Initially, participants were asked about the perceived presence of each ESs in the region, using a dichotomous scheme (0 = not present; 1 = present). They were then asked to prioritize five services they considered most relevant and evaluate them using a five‐point Likert scale: (1) very important; (2) important; (3) moderately important; (4) medium‐low importance; (5) low importance.

The Likert scale enabled the assignment of ordinal scores to each ESs, thereby distinguishing between favorable and unfavorable perceptions (Astudillo and Chevez 2021). This methodology is particularly suitable for socio‐environmental studies, as it captures intangible aspects such as cultural values, attitudes, and perceptions of well‐being (García‐Vázquez et al. 2025). Furthermore, the scale provides a balance between simplicity of application and analytical depth, facilitating comparisons across different educational, social, and cultural groups.

### 2.5 Statistical Analysis

The data obtained through the surveys was organized and processed in a database using Microsoft Excel (2025) version 16.94 (25020927). Subsequently, statistical analyses were performed with the Statistical Package for Social Sciences (SPSS) (2023) Statistics version 29.0.0.0 (241).

The analysis was structured in two complementary phases: a descriptive stage and an inferential stage. In the first phase, the frequency of mentions for each ESs (1 = present) was calculated by dividing the number of positive responses by the total number of respondents and subsequently averaging across the MEA (2005) categories.

In the second phase, an inferential analysis was applied to explore the relationship between socioeconomic variables and the prioritization of ESs. Since the ratings were recorded using a Likert ordinal scale and the data did not meet the assumptions of normality, the non‐parametric Kruskal‐Wallis test was used. This technique made it possible to identify statistically significant differences between the valuation of the ESs and the educational level of the respondents $\left(p<0.05\right)$. The other variables analyzed did not show statistically significant differences across the ESs evaluated.

Therefore, only the variable educational level was considered for the subsequent analyses. This result is consistent with previous studies that highlight the influence of educational level on the perception and valuation of ESs (Trujillo et al. 2023; Bhatt et al. 2024; Li et al. 2025). In this regard, Ge et al. (2024) argue that educational level is a determining factor in public perception and ecosystem valuation.

On the basis of ESs that showed significant differences according to educational level, a Principal Component Analysis (PCA) was conducted to reduce the dataset dimensionality and visualize the latent structures between the variables. The PCA transformed the original variables into a new set of principal components that explained the highest possible proportion of variance (Greenacre et al. 2022).

Prior to the application of the PCA and following Kherif and Latypova (2020) methodological guidelines, the data were normalized to ensure comparability. The adequacy of the model was verified by means of the Kaiser‐Meyer‐Olkin (KMO) measure and Bartlett's test of sphericity, thus ensuring the relevance of the factor analysis.

Finally, the PCA made it possible to graphically represent the distribution of educational levels in the principal component space, revealing structural associations between user profiles and prioritized ESs. Taking these findings as a reference, we formulated and designed differentiated strategies aimed at the design of adaptive public policies capable of prioritizing the ESs most valued by the different users' educational profiles.

It is important to acknowledge that the information was derived from self‐reported perceptions, which may introduce biases in responses, particularly in contexts where ESs are not easily tangible.

## 3 Results

**FIGURE 1:**
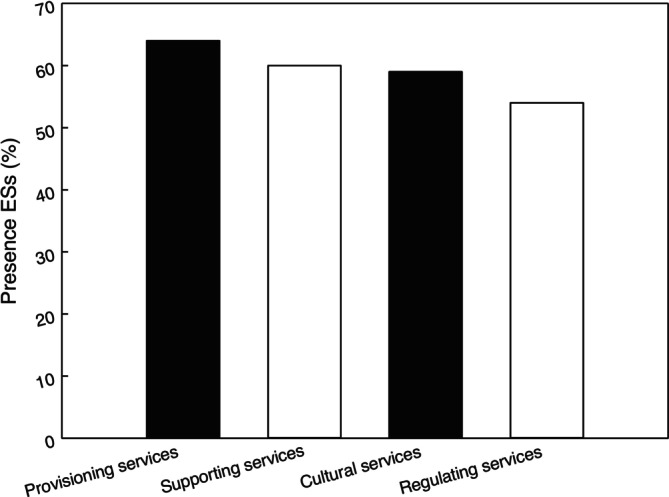
Presence of the different ESs in the area. This indicator was calculated by dividing the number of mentions of each service by the total number of respondents. The average values were then grouped according to the typology proposed by MEA (2005): provisioning, regulating, cultural, and supporting services.

Initially, the ES's presence in the study area was evaluated, confirming that all types of services are present in the territory (Figure 1). Provisioning services (64%) are the most prevalent in the area studied, closely followed by supporting services (60%) and cultural services (59%). On the other hand, regulating services show a lower presence (54%), although they still represent a significant percentage.

This suggests a remarkable strength in the region's capacity to provide tangible resources, as well as to maintain fundamental ecological processes and provide recreational or spiritual benefits.

### 3.1 Valuation of ESs Using Kruskal–Wallis Analysis

The relationship between socioeconomic variables and ESs was jointly analyzed, and it was found that the respondents' education level had a statistically significant influence. The statistical differences in the evaluation of various ESs priorities confirmed that education level acts as a differentiating factor in the way users value them (Table 2).

**TABLE 2:** Statistically significant variables with the grouping variable “Education level”.

Variable	Chi‐square	Degrees of freedom	*p* *
Priority of genetic materials	28.939	4	0.000
Priority of biochemical resource production	12.111	4	0.017
Priority of carbon sequestration and storage	18.116	4	0.001
Priority of extreme event moderation	11.561	4	0.021
Priority of spiritual value	17.067	4	0.002
Priority of historical value	17.534	4	0.002
Priority of educational value	10.036	4	0.041
Priority of habitat and breeding place	9.659	4	0.047

* Statistical significance *p* ≤ 0.05.

The priority of genetic materials suggests that people with a higher education level perceive biodiversity and genetic conservation as key factors in ecosystem management. Similarly, the high rating of the priority of carbon sequestration and storage showed that people with more education are more aware of the role of ecosystems in mitigating climate change.

In contrast, at lower educational levels, the priority of educational value and habitat and breeding place showed a greater association, indicating that respondents with a lower educational level give greater relevance to cultural services and supporting services.

Therefore, the findings showed that education level is a determining factor in the perception of the importance of ESs, especially regarding biotic conservation, carbon storage, and cultural valuation of the ecosystem. These results offer relevant contributions for the design of differentiated strategies and the formulation of sustainable conservation policies.

### 3.2 Valuation of ESs Through the PCA

The adequacy of the PCA was assessed using the KMO measure of sampling adequacy, which yielded a value of 0.817. This result indicated a high degree of adequacy for the factor analysis. Additionally, Bartlett's test of sphericity, which yielded a *p*‐value less than 0.000, confirmed that the variables were correlated, supporting the application of PCA (as shown in Table 3).

**TABLE 3:** KMO and Bartlett's test.

Measure	Value
KMO measure of sampling adequacy	0.817
Bartlett's test sphericity	
Approximate Chi‐square	261.700
Degrees of freedom	28
Value of p	<0.000

The criterion of eigenvalues greater than 1 (Kaiser's criterion) was used to select the components. The first component explained 51.175% of the variance and the second component explained 14.414%. Thus, the total variance explained is given by the two components, with a value of 65.588% (Table 4).

**TABLE 4:** Total variance explained for the PCA.

Component*	Initial eigenvalues			Sum of squared saturations of the extraction	
	Total	Variance (%)	Cumulative (%)	Total	Variance (%)
1	4.094	51.175	51.175	4.094	51.175
2	1.153	14.414	65.588	1.153	14.414
3	0.847	10.588	76.177		
4	0.728	9.099	85.276		
5	0.506	6.326	91.602		
6	0.282	3.520	95.123		
7	0.233	2.915	98.038		
8	0.157	1.962	100.000		

* 1. Production of biochemical resources; 2. Genetic materials; 3. Carbon sequestration and storage; 4. Moderation of extreme phenomena; 5. Spiritual value; 6. Educational value; 7. Historical value; 8. Habitat and breeding place.

The analysis of the principal component matrix allowed us to identify two structural clusters of ESs (Table 5). The first component presented high factor loads in the moderation of extreme phenomena (0.896), carbon sequestration and storage (0.867), genetic materials (0.854), and spiritual values (0.789); the second component showed a greater association with ESs of educational value (0.795), historical value (0.366), and habitat and breeding place (0.236).

**TABLE 5:** Principal component matrix.

Variable	Component	
	1	2
Biochemical resources production	0.537	−0.468
Genetic materials	0.854	−0.244
Carbon sequestration and storage	0.867	−0.192
Extreme event moderation	0.896	0.085
Spiritual value	0.789	−0.093
Educational value	0.295	0.795
Historical value	0.686	0.366
Habitat and breeding place	0.584	0.236

**FIGURE 2:**
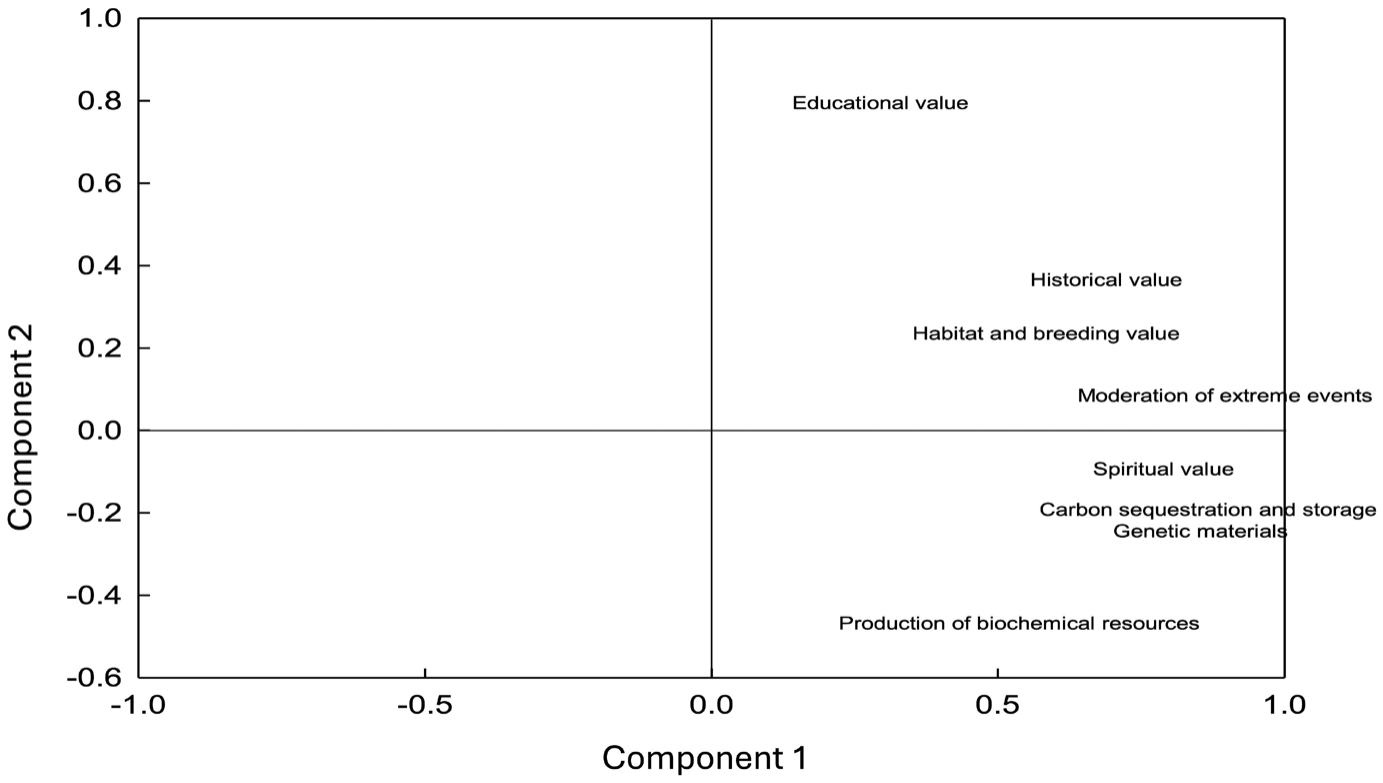
Principal components plot. The diagram identifies patterns of ESs prioritization among respondents. “Component” refers to the new statistical dimensions derived from the PCA, which groups ESs based on their observed correlations.

The PCA identified two key dimensions in the understanding and conservation of natural resources, which are represented in the factor space in Figure 2.

It was confirmed that the variables associated with provisioning and regulating ESs, such as carbon sequestration, genetic materials, and moderation of extreme events, have a higher factor load in component 1. On the other hand, variables related to cultural services and supporting services, such as educational value, historical value, and habitat, are predominantly represented in component 2.

It should be emphasized that educational value occupies a prominent position in the axis of component 2, which suggests its central role as a structuring element in this dimension.

Although the PCA extracted two differentiated dimensions, in practice these are not entirely independent. Ecosystem function is linked to the four categories of ESs (provisioning, regulating, cultural, and supporting), since the capacity of an ecosystem to generate recreational, spiritual, or identity‐related benefits depends directly on its ecological integrity.

This finding represents not only a first step toward understanding how territory is valued but also a basis for designing strategies that recognize this heterogeneity while also considering these intrinsic relationships.

**FIGURE 3:**
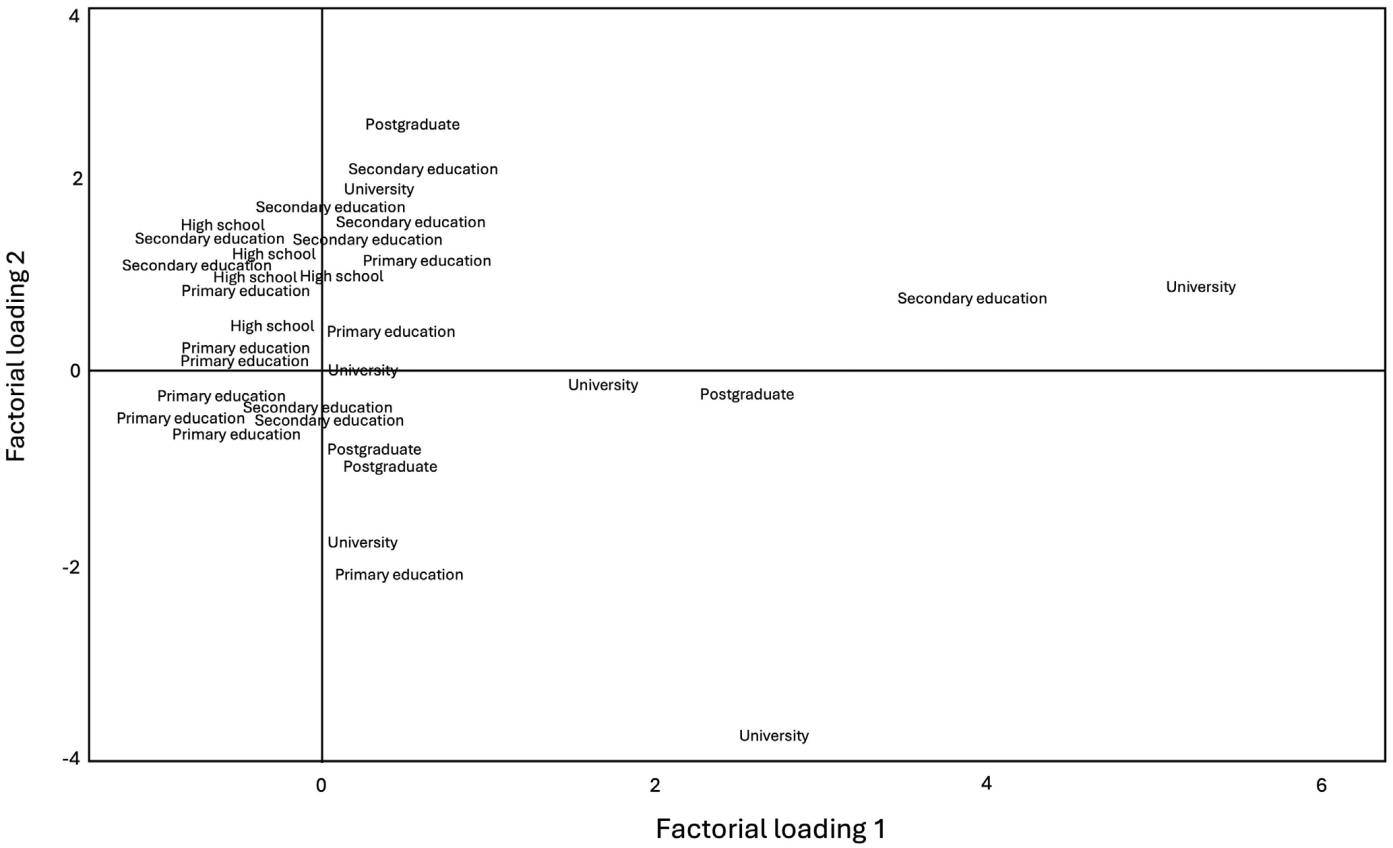
Classification of principal components by educational level with factor loads. The diagram shows the classification of ESs according to the respondents' educational levels. This visualization highlights how different educational levels are associated with the two dimensions obtained from the PCA.

Figure 3 shows the distribution of the different educational levels in the space defined by the principal components. A trend is observed in which participants with a higher level of education (university and postgraduate) are grouped more toward component 1 (factor load 1), which is related to ecological functionality (provisioning and regulating services).

On the other hand, participants with a lower education level (primary education and secondary education) tend to be placed in component 2, which is more related to cultural and population support aspects, such as historical value and habitat (cultural services and supporting services).

The dispersion in the figure also suggests that intermediate educational levels, such as secondary education and high school, have a heterogeneous distribution, showing a combination of interests in the different types of ESs (provisioning, regulating, cultural, and supporting).

The research results indicated that educational level not only influences the prioritization of ESs but also reflects an intricate interplay of social, economic, and cultural factors mediating this relationship. For instance, individuals with higher education levels tend to clearly recognize the importance of provisioning and regulating services, which may be linked to broader access to scientific information and global environmental frameworks.

In contrast, those with lower education levels prioritize cultural and supporting services, partly due to their direct dependence on natural resources for productive and subsistence activities, reflecting immediate economic interests. Their valuation also underscores the significance of traditional practices and the intrinsic cultural relationships embedded within the territory.

This interaction between education, socioeconomic conditions, and cultural capital is crucial for understanding differences in ESs valuation and provides essential input for the conservation policies design that acknowledges the social heterogeneity of the territory.

### 3.3 Proposal of Policies Based on the Prioritization of Users and the Analysis of the ESs

In accordance with the pattern of the valuation of the ESs in the area, it is proposed to design differentiated conservation strategies; this proposal is based on a direct alignment with the valuation of the users.

For users with university and postgraduate educational levels, policies should focus on the conservation of the ecosystem functions of natural capital, such as regulating and provisioning functions. Likewise, they should focus on the mitigation of climate change.

A differentiated design of payments for environmental services is suggested. The design of the environmental policy should outline the prioritization of carbon sequestration and biodiversity conservation through economic incentives. Likewise, it should outline ecological zoning through the analysis of priority conservation areas.

On the other hand, the promotion and production of genetic and biochemical resources through incentives for productive economic sectors is emphasized. A precise line of action is the research of species with value in the market and their positioning with agroecological production in the area.

For users with an intermediate education level (secondary education and high school). Since this group has mixed interests, policies must integrate conservation and sociocultural benefits of the ecosystem. For this reason, educational programs that focus on the sustainable use of natural resources and the generation of green employment are a priority.

Subsidies or certification programs for producers who adopt regenerative methods also become relevant. The development of infrastructure for community‐based ecotourism activities through cultural valuation is an effective strategy for the area.

The users with a lower education level (primary education and secondary education) have a greater inclination for cultural services, cultural valorization, and supporting services. The strategies that should be established are framed in the conservation and cultural appropriation of ecosystems.

The creation of sustainable business models and protected areas through integrated management is recommended. They should establish community participation through the integration of traditional knowledge. Similarly, the creation of legal figures that recognize the role of communities in the protection of ecosystems is suggested.

Finally, to strengthen the protection and financing of cultural ESs, it is necessary to establish institutions that study and quantify their economic value.

In general, for these proposals to be effective, various studies are required to complement and support the argumentative basis of the projected policies.

## 4 Discussion

### 4.1 ESs and Socioeconomic Factors

Initially, it was shown that all types of ESs are present in the area; a higher prevalence of provisioning services was noted, followed by supporting, cultural, and regulating services. These findings are consistent with what was reported by Aryal et al. (2021), who highlight that in local communities, ESs that provide immediate and tangible benefits are typically valued more highly.

A significant relationship was also identified between education level and ESs prioritization. This finding aligns with international research showing how education influences environmental perception. For example, studies conducted in Europe and Asia have indicated that higher education levels are associated with greater valuation of regulating services, largely due to increased access to environmental information and higher ecological awareness (Janeczko et al. 2023; Li et al. 2025).

Trujillo et al. (2023), in their analysis of a group composed of researchers, professors, and undergraduate and graduate students, found that regulating and provisioning services were most highly valued, as they are essential both for ecological balance and for sustaining livelihoods.

On the other hand, it has been shown that local communities with lower educational levels tend to value cultural and supporting ESs primarily for their role as spaces of social cohesion and their importance in agricultural biodiversity (Géant et al. 2024). However, they do not always consider them to be the most important ESs.

In this regard, several authors highlight that the educational and historical values of cultural services reflect the role of territories as essential spaces of identity (Ahrens et al. 2025). In contrast, in urban contexts, the valuation of cultural services is often linked to recreational and tourism‐related activities (Li et al. 2025).

These differences underscore the influence of cultural and geographical contexts in shaping environmental priorities and suggest that education does not operate in isolation, but rather interacts with social, economic, and cultural factors (García‐Llorente et al. 2020).

Therefore, education level should not be analyzed independently. Other factors, such as gender, age, occupation, and socioeconomic context, have also been shown to influence ESs' perception and prioritization (Janeczko et al. 2023; Pinto et al. 2023; Trujillo et al. 2023; Bhatt et al. 2024; Li et al. 2025). This evidence highlights the need for future studies to incorporate broader multivariate approaches to disentangle the interplay between education and other social determinants in ESs' valuation.

### 4.2 Valuation of ESs in Semi‐Arid Contexts and in the Study Area

In semi‐arid contexts, the valuation of ESs represents a key tool to guide land use, promoting sustainable development, and ensuring ecological protection (Jia et al. 2021). Several studies have documented that, in these environments, social preferences tend to focus primarily on provisioning, regulating, and cultural services (Zabala et al. 2021).

Silva et al. (2021) confirmed this trend in a study conducted in arid and semi‐arid ecosystems in southern Spain, where 24 ESs were identified within the framework of an ecological restoration process. Their results showed that the most highly valued services were provisioning (7), regulating (7), and cultural (6) services (Silva et al. 2021).

In the specific case of the Laguna de Bustillos basin, land‐use changes have played a decisive role in shaping ecological dynamics. Valencia‐Gaspar et al. (2024) reported that between 1974 and 2016 the region experienced a marked expansion of agriculture and urban areas, accompanied by significant losses of grasslands, oak–pine and pine forests, as well as water bodies. These transformations have directly altered both the provision and the perception of ESs in the region.

The basin is recognized as a natural and agronomic asset of fundamental importance for regional development. However, inadequate management of human activities has led the lagoon to receive flows of sediments and wastewater, deteriorating both water quality and local living conditions (Medina‐Esparza et al. 2025). Beyond its ecological value, Laguna de Bustillos basin also constitutes a cultural, economic, and social landmark in the agricultural valley of Cuauhtémoc, Chihuahua (Reyes et al. 2024).

Despite its importance, no prior studies have specifically focused on the valuation of ESs in this area. Therefore, the present research provides an initial approach that offers scientific evidence on the importance of integrating social perceptions into the management and conservation of a strategic ecosystem for Mexico.

### 4.3 Importance of ESs

The ecological, economic, and social complexity of territories highlights the need to deepen research focused on the valuation of ESs (Sánchez et al. 2023). The present study aligns with the United Nations Decade on Ecosystem Restoration (2021–2030), which promotes collaborative actions to restore degraded landscapes and strengthen socio‐ecological resilience (Waltham et al. 2020).

Nevertheless, these efforts unfold in a context marked by critical pressures such as deforestation, habitat fragmentation, and climate change—factors that directly affect the structure and functioning of ecosystems (Mieles‐Giler et al. 2024). At the same time, the Sustainable Development Goals (SDGs) face challenges arising from trade‐offs between environmental protection and human well‐being (Yang et al. 2020).

In this context, ESs‐based solutions emerge as a promising approach, as they enable the articulation of socio‐economic development with the conservation of natural capital (Yang et al. 2020). To make ESs a practical and effective strategy, it's crucial to combine participatory governance, territorial planning, and the efficient use of natural resources, all within a unified framework (Wang et al. 2021).

Such integrative approaches are particularly relevant in territories with complex socio‐ecological dynamics, as they foster collaboration among local communities, scientists, and authorities, thereby promoting more inclusive, adaptive, and sustainable environmental management processes (Leguia‐Cruz et al. 2024).

### 4.4 Use of Methodological Tools

The results of the Kruskal–Wallis analysis revealed statistically significant differences in the perception of different ESs according to the respondents' education level. This association has been extensively documented by previous research, such as those by García‐Llorente et al. (2020), Ge et al. (2024), and Granobles et al. (2024), which coincide in pointing out that education is a determining factor in the way people value the benefits provided by nature. However, as noted earlier, education should be understood as part of a broader mosaic of variables necessary to fully capture how individuals perceive and prioritize ESs.

On the other hand, by identifying two key structural dimensions in the valuation of ESs, the PCA is grouped and aligned with current theoretical approaches that suggest that ecosystem valuations should be understood from an interdisciplinary perspective (Castellar et al. 2021; Rosa‐Velázquez and Ruiz‐Luna 2023; Caballero‐López et al. 2024).

In previous studies, Zhang, Wang, et al. (2021) identified two principal components that explained the variability of a wetland system, one linked to socioeconomic factors and the other related to meteorological and geographical conditions. These findings are consistent with the factor structure obtained in the present research, where clear clusters between components of ecological functionality and socioeconomic variables can be observed.

From a management and policy‐making perspective, it is essential to account for these differences to design differentiated and collaborative policies.

### 4.5 Design of Differentiated and Collaborative Policies

The results of the study support the proposal for the design of differentiated conservation policies according to the needs and priorities of users. In agreement with Trujillo et al. (2023) and García‐Vázquez et al. (2025) it is suggested that one way to improve the effectiveness of programs and policies is through the identification of the target user group and their natural resources.

In this line, ESs have demonstrated potential uses in multiple time and space scenarios (Chen et al. 2023). Nevertheless, a comparative analysis of the drivers of ESs in each region is necessary to optimize effective policies (Fang et al. 2021). As for their potential uses, these range from increasing public awareness to allowing for detailed analysis of different options and scenarios (Chen et al. 2023).

At the same time, conservation policies must consider the impacts related to climate change and land use (Li et al. 2021). To this end, it is crucial to integrate different valuation methods, both monetary and non‐monetary, that allow the interaction between the different components of the ESs to be synergistically assessed (Romanazzi et al. 2023).

In the case of this research, by determining that some key strategies have to focus on the conservation of specific ecosystem functions and include mechanisms such as payments for environmental services and ecological zoning. They are aligned with what Luo et al. (2020) pointed out, who indicated that, for a healthy development of ecosystems, the protection and increase of ecological areas are required. For their part, Su et al. (2020) and Zhang et al. (2020) showed that monetization of ESs can increase public awareness of their importance for social development.

Other initiatives are based on environmental education and incentives for agroecological practices. Similar studies have confirmed that environmental education is key to improving the quality of ecosystems (Ge et al. 2024). Educational programs designed for local communities can raise awareness about ESs and encourage their conservation (Khachatryan 2024). Likewise, programs aimed at young students have the potential to generate significant changes since they are receptive to new ideas and to forge pro‐environmental behaviors (Li et al. 2025).

Agroecological practices have also been identified as an effective strategy for the improvement of the diversity of cultivated species, which contributes to the sustainable management of ecosystems and the optimization of both agricultural yield and biodiversity and ESs (Beillouin et al. 2021).

As for policies related to cultural valuation and the creation of sustainable business models and protected areas, they have proven to be relevant initiatives for strengthening and conservation processes, achieving significant progress (Narváez et al. 2023). The integration of ecological management with community participation and inter‐institutional coordination is transforming the conservation paradigm and promoting effective ecosystem management (González et al. 2024).

Although the relationship between ESs and human well‐being has been recognized and incorporated as a basis for policymaking, a thorough review of this relationship and its implications is still required (Zhou et al. 2024). It is essential to identify appropriate policy indicators and incorporate them into the analysis of ESs (Pan et al. 2021).

## 5 Study Limitations

This study acknowledges certain limitations. First, the analysis was based on a localized sample, which restricts the generalization of the findings to other socio‐ecological contexts. Second, although the influence of educational level on the prioritization of ESs was identified, the study did not explore in depth the specific mechanisms through which education interacts with social, economic, and cultural factors to shape such valuations.

Therefore, it is recommended to conduct complementary research that allows for a more detailed analysis of the processes through which education influences the perception and prioritization of ESs, as well as to incorporate additional socioeconomic variables that can enrich the analysis. Likewise, these studies should be complemented with in‐depth interviews, participatory mapping, and longitudinal techniques to evaluate changes in perceptions over time.

Such efforts would strengthen the validity of the findings and provide more robust inputs for the design of differentiated and adaptive public policies.

## 6 Conclusions

The study allows us to observe the existing relationships between the prioritization of ESs and education; this relationship shows a substantial contribution to the design of differentiated environmental conservation strategies. The specific results indicate that provisioning services were the most highly valued in the area.

Moreover, individuals with higher educational levels tend to prioritize the functional aspects of the ecosystem, such as provisioning and regulating services, whereas those with lower educational attainment place greater importance on cultural and supporting services, such as historical value and habitat.

The analysis based on the prioritization of users, in addition to recognizing the generation of effective strategies, is a tool that, through proper management, can maximize the conservation of ESs in the long term.

Placing these findings in the context of current environmental challenges, such as biodiversity loss and pressures on natural resources, underscores their relevance. For instance, integrating the perspectives of those who prioritize functional services with those who value cultural services enables the development of more inclusive strategies. This, in turn, requires participatory governance spaces in which communities, institutions, and resource users collectively define priorities and responsibilities.

However, assessing the actual impact requires complementary analyses that help to better understand the relationships between social perceptions, environmental valuation, and the effectiveness of differentiated public policies. For that reason, it is recommended to continue conducting studies in this area to strengthen the understanding of these dynamics.

## Author Contributions

**Blanca Isabel Sánchez Toledano:** conceptualization (lead), investigation (lead), supervision (lead). **Mercedes Borja Bravo:** conceptualization (supporting), funding acquisition (lead), project administration (lead), resources (supporting). **Rafael García Vázquez:** formal analysis (lead), methodology (lead), software (lead), writing – original draft (lead). **Marco Andrés López Santiago:** data curation (lead), supervision (lead), validation (lead), writing – review and editing (lead).

## Conflicts of Interest

The authors declare no conflicts of interest.

## Supporting information


**Data S1:** ece372344‐sup‐0001‐Supinfo01.xlsx.

**Data S2:** ece372344‐sup‐0002‐Supinfo02.docx.

## Data Availability

All required data is uploaded as Supporting Information. They are also available in the data repository: https://docs.google.com/spreadsheets/d/1rd5Rphg_HfVhckAltLxS4m8ZFgAgjQW7/edit?usp=share_link&ouid=104117707354989824776&rtpof=true&sd=true.
