# Supplementary material for: Prioritization of Users in Ecosystem Service Valuation: Implications for the Design of Differentiated Conservation Policies
Source: Ecol Evol. 2025 Oct 17;15(10):e72344. doi: 10.1002/ece3.72344 (PMC12531590; doi:10.1002/ece3.72344)
Supplement: Supplementary file 2 — Data S2: ece372344‐sup‐0002‐Supinfo02.docx. [file ECE3-15-e72344-s002.docx]

The following survey is part of a scientific research project conducted by the National Institute of Forestry, Agricultural and Livestock Research (INIFAP). Its purpose is to gather information about the ecosystem services provided by the Bustillos Lagoon Watershed.

SURVEY 1: IDENTIFICATION AND SELECTION OF ECOSYSTEM SERVICES

**ID Number: _______________________________**

**Interviewer: _______________________________**

**1. Name:** _______________________________________________

**2. Age:** __________years **3. Gender:** Male____ Female____

**4. Educational level:**

| **Elementary** | **Secondary** | **High School** | **University** | **Postgraduate** |
| --- | --- | --- | --- | --- |
|  |  |  |  |  |

**5. Locality:** _____________________________________________________________

**6.Municipality :** ______________________ **7. State:** ____________________

**8. Employment (you may select more than one):**

|  | Farmer |  | Municipal public servant |
| --- | --- | --- | --- |
|  |  |  |  |
|  | Rancher |  | State public servant |
|  |  |  |  |
|  | Businessperson |  | Federal public servant |
|  |  |  |  |
|  | Employee |  | Business owner |

**9. Instructions**

On the following page, you will find a table listing potential ecosystem services provided by the Laguna de Bustillos watershed reserve.

First, complete the identification information at the top of the page.

Then, in the “Existence Confirmation” column, mark with an X if you recognize the existence of the service described in each row (you may check as many as you consider applicable).

In the “Priority” column, select the five (5) services that you consider most important and rank them from 1 to 5, with 1 being the highest priority.

| **Ecosystem Services** | | **Description** | **Presence Confirmation** | **Priority** |
| --- | --- | --- | --- | --- |
| **Provisioning Services** | **Food production** | Vegetables-fruits production and hunting and fishing areas. |  |  |
|  | **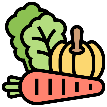** |  |  |  |
|  | **Ornamental species production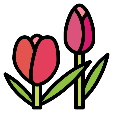** | Non-edible plant and animal species production for recreational or ornamental purposes. |  |  |
|  | 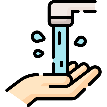**Water storage and retention** | Rainwater retention in aquifers and provision of water for irrigation or drinking. |  |  |
|  | **Production of raw materials and fuels**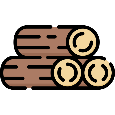 | Production of wood, firewood, peat, fibers, forage, composite materials, biofuels or cultural artifacts. |  |  |
|  | **Biochemical resource production**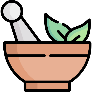 | Medicinal plants and other products with pharmaceutical importance. |  |  |
|  | **Genetic materials**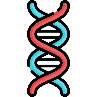 | Unique plant genotypes and local species resistant to phytopathogens. |  |  |
| **Regulating services** | 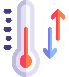 **Climate regulation** | Regulation of air quality, temperature and precipitation. Dust particles capture. |  |  |
|  | 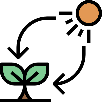**Carbon sequestration and storage** | Regulation of greenhouse gases and other pollutants. |  |  |
|  | 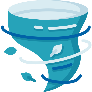**Water flow regulation** | Groundwater recharge/discharge and water purification. |  |  |
|  | 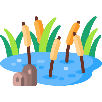**Extreme event moderation** | Flood control and storm protection. |  |  |
|  | **Erosion control**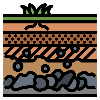 | Soil retention and prevention of soil structural degradation. |  |  |
|  | 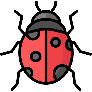**Biological regulation** | Habitat for pollinators including insects, birds, and bats. Pest control. |  |  |

| **Cultural services** | **Recreational activities**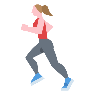 | Support for physical and mental health, opportunities for tourism and leisure activities (tours, zip-lining, etc.). |  |  |
| --- | --- | --- | --- | --- |
|  | 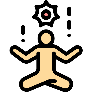**Spiritual value** | Spiritual sense of belonging, traditional knowledge and customs. |  |  |
|  | 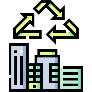**Educational value** | Opportunities for formal and informal education and training. |  |  |
|  | 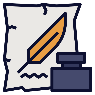**Historical value** | Archeological sites (cave paintings, mammoth remains). |  |  |
|  | **Aesthetic value**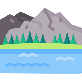 | Nature and landscapes appreciation. |  |  |
|  | **Cultural heritage and identity** 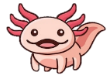 | Sense of place and belonging. |  |  |
| **Supporting services** | **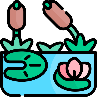Habitat and breeding place** | Habitat for resident and migratory species (birds, butterflies). |  |  |
|  | **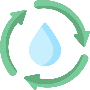** **Nutrient cycling** | Storage, recycling, processing and acquisition of nutrients. |  |  |
|  | **Primary productivity** 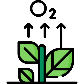 | Oxygen production by plants and phytoplankton. |  |  |

**We greatly appreciate your participation.**
